# Experimental Physiology

**Published:** 1868-08-15

**Authors:** W. Laborde

**Affiliations:** Paris


### CHICAGO MEDICAL JOURNAL.

*Vol. XXV.—AUGUST* 15, 1868.—*No.* 16.

### BIOLOGICAL SOCIETY--EXPERIMENTAL PHYSIOLOGY.

*Report upon the Physiological Action of Bromide of Potassium, established by Experiments upon Animals. By Dr.* W. LABORDE, *Paris.*

TRANSLATED EXPRESSLY FOR THIS JOURNAL, BY WALTER HAY, M.D., ASSOCIATE EDITOR.

IN 1864, at the time when the initiative of my regretted preceptor, M. le Dr. Debout had just awakened in France attention to the *Bromide of Potassium*, certain experiments made upon myself with a view to determine the physiological ction of this substance upon man, demonstrated to me that the large doses, extolled by M. le Dr. Puche, physician to L'Hospital du Midi, and by his internes MM. Rames and Huette were far from possessing all the innocence, which they were induced to attribute to them. I resolved thence, in order to determine the physiological and toxic effects of this compound, to submit it to the test of experiment upon animals. I believe that I have obtained upon this subject results which deserve some attention, and which I desire to submit to the appreciation of the society.

I have prepared for the purpose two of my principal experiments, of which I shall make my colleagues the witnesses. I shall thence confine myself for the moment to the deduction from these experiments, and the recording of essential conclusions which are implicitly contained therein.

Exp. 1.—Into a vigorous frog (*Rana Viridis*), I introduced through the interdigital membrane of each paw 15 centigrammes of *Bromide of Potassium* (in all 30 centigrammes). For this purpose I make use of a process which I have already described; that is to say, that after having previously stretched the interdigital membrane I have applied the crystals of *Bromide of Potassium* in their natural state, taking care to pour upon these crystals some drops of water, in order to facilitate their solution and absorption; this is very rapid, moreover, and five minutes after the commencement of the experiment, the effects of the *Bromide* began to manifest themselves. The animal left to itself, executes, in its place, spontaneous and apparently convulsive movements; then the posterior paws remain inert and extended, presenting a certain flaccidity which does not seem any longer to permit the tonic flexion of the limb, which characterizes the normal flexion of the limb when in repose.

If one or the other of these paws is excited either by simply pinching, or by pricking, or by the aid of the electric current, the animal reacts, at first very feebly against these irritants, and soon, that is to say, after a quarter of an hour or twenty minutes, it no longer reacts at all, whatever may be the degree of irritation, which may be carried even to tearing, to crushing, or to section.

The same state of affairs is observed in the anterior members, but at a somewhat more advanced period of the intoxication it is seen to extend itself likewise to the two eyes, for the irritation of the cornea and the sclerotic provokes, only with great difficulty, the closure of the lids.

However, at the very moment when the phenomena of reactional impossibility commence to manifest themselves, the animal executes, under my observation, certain partial spontaneous movements several times repeated.

The movements of the flank, which at the beginning presented a noticeable acceleration, underwent, soon afterward, a progressive retardation. They ceased completely three-quarters of an hour after the commencement of the experiment. At this moment the collapse is complete, and the animal is in a state of apparent death; every motor or voluntary manifestation is abolished. The frog being now turned over violently upon its back, reacts only by certain fibrillous trembling (*tressailements fibrillaires*), which are habitually the last manifestations of a frog which is definitely given up to death.

If we now open the chest, we observe that the heart continues to perform its functions rythmically, if not with the normal number of pulsations. These continue, however, although decreasing progressively for nearly two hours.

Exp. II.—In a frog of medium strength, but very lively, we have, as a preliminary step, ligated the artery of the left paw, taking care not to include the nerve in this ligature. We have next placed upon the interdigital membrane of the posterior paw of the right side, 15 centigrammes of the *Bromide of Potassium*, moistening the crystals with some drops of water. Absorption has been rapid since it has been accomplished in less than five minutes; nevertheless the action of the chemical agent is slower than usual in manifesting itself, by reason, probably, of the inferiority of the dose, which, however, has appeared to us sufficient for the subject of the experiment.

The frog, left to itself, manifested already, as we perceive, a certain degree of agitation with evident rigidity of the dorsal columns; it effects some very vigorous leaps, then is only able to move its paws on the spot. Soon the right posterior paw remains relaxed and almost inert, whilst the left paw (protected by the ligature from the action of the *Bromide*), preserves its tonic flexion; but this last does not long delay permitting itself to be placed, like the other, without reaction, in a state of passive elongation. The animal, nevertheless, performs, at intervals, energetic spontaneous movements.

About twenty minutes have passed since the absorption of the substance. If the extremity of one or the other posterior paws be pinched, the animal at first only reacts very feebly upon one side equally as upon the other. Soon, however, a bistoury may be plunged successively in the two members, in such a manner as to traverse them thoroughly, without producing the least reactional movement neither to the right nor to the left; the same condition exists in the anterior extremities. At the same time, the movement of the flank is perceived to lessen progressively. The animal is prostrated, and yet he originates from time to time voluntarily movements in the whole of his members. If the nerve of the left paw, which is exposed by the side of the litigated artery, be touched, or especially be pinched or pricked, it effects vigorous motor efforts in the limb of that side—the same thing occurs exactly in the paw of the opposite side.

To prevent any possibility of voluntary impulse, we rapidly decapitate the frog. Universal tremors follow this operation. When tranquility is re-established in the trunk of the animal, every species of irritation of the peripheral organs, whatever may be its violence, extorts no reactionál response; the denuded nerve, directly excited, continues to provoke muscular contractions.

Moreover, the spinal marrow, touched or irritated in its canal, with a very fine and very sharp stylet, affords at first very distinct evidences of the preservation of its normal excitability, for all the limbs of the animal are simultaneously agitated by violent and jerking movements exactly resembling those of a wooden jumping-jack, when its string is pulled; if the irritation is maintained during a certain period, the limbs remain in a state of tonic, and, as it were, a tetanic contraction; but the phenomenon exhausts itself soon, without the possibility of being reproduced. The normal excitability of the cord hardly exists for a quarter of an hour under these circumstances, and it appears to diminish progressively from below upwards, that is to say, the excitability of the cords persists a longer time in its superior regions, in which it still gives evidence of its existence after it appears to be completely extinct in the lumbar.

From these experimental facts, which, repeated a great number of times, have given constant results, we are permitted to conclude:

1. That the *Bromide of Potassium*, introduced into the animal organism by the natural physiological channels, exercises a predominant action over the nervous system in general, and more particularly over the *sensori-motors* of the reflex order by implicating simultaneously the central organ of the elaboration of these phenomena, that is to say, the spinal cord, and the peripheral sensitive nervous expansions.

2. That this action is reflected only secondarily upon the organs and the functions of *voluntary motion* (the brain and nervous conductibility and mobility).

3. That muscular contractility appears to be respected by the *Bromide of Potassium*, and the contractility of the heart, in particular, persists after the manifestations which betray the influence of this salt upon the other organs and functions are produced; the heart, in fact, is the *ultimum moriens* in the bromic intoxication; from which it results, that it would have been entirely erroneous to consider the *Bromide of Potassium* as a cardiac poison.

---

*Experimental Studies upon the Physiological Action of Bromide of Potassium.* *By* MARTIN DAMOURETTE and PELVET.

The experiments of MM. Martin Damourette and Pelvet have been made upon the frog, the rabbit, and birds (swallows, pigeons and magpies); the phenomena produced have been the same with different animals, but it is upon the rabbit that they can be most easily analyzed. Moreover, the results obtained upon batrachians are those which the authors report in a more detailed manner.

*Experiments upon Frogs.* The processes employed have been gastric ingestion, application to the extremity of a paw, and injection into the hypodermic cellular, by means of the syringe of Baraz. The injections have been made in three principal regions: in the groin, in the axilla, and in the back, in the flank, and under the sternum. The medium dose resulting frequently, although not constantly, in the death of the animal, were from 3 to 5 centigrammes. Moreover, the authors employed weak doses, followed always by recovery (from 5 to 25 milligrammes), and strong doses invariably faalt (from 5 to 10 centigrammes).

A. *Effects of small doses.* The frog becomes quiet; it seems seems to forget to breathe. By the least stimulation it begins to breathe and to jump; then it relapses into the same calm. The duration of the intoxication varies from six to twenty-four hours.

B. *Effects of medium doses.* Pain at the level of the place of injection, trembling in the neighboring muscles, then enfeeblement of motion, and of sensibility in the vicinity at the end of five or ten minutes, and generally at the end of twenty to forty minutes. It is important to distinguish here the effects of imbibition for those produced by absorption. Muscular irritability is extinguished for a few minutes in the muscles of the regions injected, whilst in the more distant muscles, and those upon which absorption only has acted, it persists after the loss of excitability of the nerves and of the cord.

The motor excitability of the nerves survives this sensibility, and that of the cord (contrary to the opinion of M. Laborde and of MM. Eulenbourg and Guttman), persists even when the nerves have lost their properties. Finally the properties of the cord are abolished in their turn.

From the beginning of the intoxication the frog slumbers. The encaphalon, therefore, undergoes the action of the *Bromide* like the rest of the nervous system. The respiratory movements cease ordinarily a little after the voluntary movements, that is to say, from ten to thirty minutes after an injection into the grain, and from five to fifteen minutes after an injection into the flank or back.

The diminution of the capillary circulation characterized by the retardation of the course of the blood in the vessels of the interdigital membrane is a constant phenomenon; it commences after about five minutes in the interdigital membrane of the injected side, where it attains its greatest intensity, extending itself thence after ten to forty minutes to the membrane of the other side, and throughout the entire capillary network; it precedes the retardation of the cardiac pulsations which supervenes from the instant that the movements become enfeebled, and the respiration arrested.

The heart is the *ultimum moriens;* it has continued to beat many hours after the spinal cord, the nerves and the muscles had ceased to respond to all irritations. MM. Eulenbourg and Guttman were, therefore, wrong in considering the *Bromide of Potassium* as a double poison—of the heart first, and afterwards of the spinal cord.

C. *Effects of strong doses.* The same spmptoms as in the preceding cases, only with more intensity and rapidity. In some cases the cessation of the heart's action supervenes prematurely, (before the loss of the excitability of the nerves and spinal cord, and muscular irritability). Moreover, congestion of the capillary network may be observed replacing the anemia, which was constant with weak and medium doses.

*Experiments upon birds and animals.*—By the subcutaneous injection into the groin, or axilla, of four grammes in the rabbit, of sixty centigrammes in the pigeon, and of ten centigrammes in the sparrow, we have—

1. Paralysis of sensation and of motion, commencing in the rejected limb, and continuing throughout.

2. Diminution of temperature. At first, of the injected part, and afterward of the whole body. Frequent and even bloody urination.

3. Respiration at first disturbed, only arrested when all the other parts are paralyzed; its suspension appeared to be the cause of death, and marks its precise moment; it ensues with the rapidity of lightning in birds.

The authors next devote themselves to a detailed analysis of *Bromium* upon each of the systems and organic apparata; and draw therefrom interesting therapeutic deductions. We refer for these to the originals.

NOTE.—Effects somewhat similar to the above are manifested upon the application of *Aqua Ammonia* to the spinal column (cutaneous surface) of *rana viridis.* The application of a few drops of the liquid is followed by vigorous motion on the part of the animal, occasioned, undoubtedly by the pain. These manifestations are succeeded by immediate and complete loss of contractility, both voluntary and reflex, in the posterior extremities, which extends itself gradually upward along the cord, involving, subsequently, the anterior members, which, in the interval, continue to manifest voluntary contractility for several minutes: respiration and circulation failing last of all. TRANSLATOR.

---

### *Experimental Physiology—Vaccine and Quinine.*

There were presented to the Academy of Sciences (Paris), at a recent meeting, two communications upon two subjects closely allied, for they are both closely allied with the study of the infinitesimal, which is, at this time, pursued with so much ardor—the nature of virus, and the antiseptic properties of certain agents of the Materia Medica.

In a work relating to microzoa and microphytes in the genesis and evolution of diseases, a work whose whose publication we have been compelled to suspend in consequence of the weekly obligations of the *Gazette Médicale*, but which we will still soon resume, we have have shown the tendency of certain minds to attribute to the presence of these microscopic beings in the blood and in the fluids of the organism, the cause and origin of virulent and zymotic affection. This opinion appears even to grow and to be recruited by new adherents, for daily abroad as well as in France, we observe the discovery of animal or vegetable parasites in the pathological products of infectious or contagious diseases.

We may state especially, since the first communication of which we have spoken refers to vaccine, that the German observers MM. Schurtz, Hallier et Zürn, have found mycrophytes in vaccina pustules. Must we conclude thence that the action of vaccine is due to the presence of parasites, just as, according to the investigations of M. Davaine, Bacteria constitute the virulent agent in carbon? This would not appear to result from the experiments, of which M. Chaveau has communicated the results to the Academy of Science.

The learned physiologist of Lyons has separated by dialysis the different elements which constitute vaccine, and has endeavored to determine, experimentally, which one among them is necessary in order to secure the successful inoculation of the vaccine. He has, therefore, isolated and inoculated separately—1st, the albuminous serosity; 2nd, the white globules; 3rd, the solid, molecular granulations suspended in the serosity.

The two first series of experiments have given negative results; the granulations alone, even when suspended in ten times their volume of water, have produced legitimate vaccine.

M. Cl. Bernard, who was charged with the presentation to the Academy of this work of M. Chauveau, added that it had been established that the virulent agent of other virus resides in the solid granulations.

What is the nature of these granulations? Would the partizans of animated pathology be authorized to recognize in them living germs, eggs or sporules? The question is not yet susceptible of solution, and demands further investigation. However, this may be, and without reference to the animist or mysterious explanation which has been given of the nature of virus, we find always presented to us, three hypotheses. Some, indeed, locate the virulent action, not in one of the elements of the liquid, but in the entire liquid, which has sustained simply isomeric modification; others attribute it to living microscopic organisms, whose presence they have discovered; and lastly, it would result from the paper of M. Chauveau, and from the confirmation which it has received from M. Claude Bernard, that it is in the solid granulations that the agent of virulence must be sought. Light upon all these points is needed.

The second communication presented to the Academy by M. Pasteur, in behalf of a professor of Bonn, is more nearly related to the order of ideas which we suggested at the commencement. It relates to the antiseptic properties of the *Salts of Quinine.* These properties must be very energetic, a proportion of one twenty thousandth of *Chlorohydrate of Quinine* will suffice to kill kolpodes and all the paramecea of an infusion. On the other hand, the *Quinine* will act upon the white globules of the blood by arresting the motion with which they are endowed, and by diminishing their number. This may be a new mark of analogy between the white globules of the blood and certain animalculæ to which they have been compared. M. Pasteur does not appear to differ widely from the opinion which considers these globules as microzoa, and it must be recognized, if this fact is generally admitted, that it would constitute by no means the least of the conquests which the doctrine of animated pathology may be permitted to hope for.

